# Loss of WISP2/CCN5 in Estrogen-Dependent MCF7 Human Breast Cancer Cells Promotes a Stem-Like Cell Phenotype

**DOI:** 10.1371/journal.pone.0087878

**Published:** 2014-02-03

**Authors:** Nathalie Ferrand, Anne Gnanapragasam, Guillaume Dorothee, Gérard Redeuilh, Annette K. Larsen, Michèle Sabbah

**Affiliations:** 1 Cancer Biology and Therapeutics, Centre de Recherche Saint-Antoine, Paris, France; 2 Immune system, Neuroinflammation and Neurodegenerative diseases, Centre de Recherche Saint-Antoine, Paris, France; 3 Institut National de la Santé et de la Recherche Médicale, Paris, France; 4 Université Pierre et Marie Curie, Paris, France; University of Birmingham, United Kingdom

## Abstract

It has been proposed that the epithelial-mesenchymal transition (EMT) in mammary epithelial cells and breast cancer cells generates stem cell features. WISP2 (Wnt-1-induced signaling protein-2) plays an important role in maintenance of the differentiated phenotype of estrogen receptor-positive breast cancer cells and loss of WISP2 is associated with EMT. We now report that loss of WISP2 in MCF7 breast cancer cells can also promote the emergence of a cancer stem-like cell phenotype characterized by high expression of CD44, increased aldehyde dehydrogenase activity and mammosphere formation. Higher levels of the stem cell markers Nanog and Oct3/4 were observed in those mammospheres. In addition we show that low-cell inoculums are capable of tumor formation in the mammary fat pad of immunodeficient mice. Gene expression analysis show an enrichment of markers linked to stem cell function such as SOX9 and IGFBP7 which is linked to TGF-β inducible, SMAD3-dependent transcription. Taken together, our data demonstrate that WISP2 loss promotes both EMT and the stem-like cell phenotype.

## Introduction

CCN5, also known as WISP2, is a ∼ 29-kDa protein which belongs to the cysteine-rich 61/connective tissue growth factor/nephroblastoma overexpressed (CCN) family [1]. This gene family has been implicated in a wide range of physiological and pathological processes including proliferation, differentiation, migration, angiogenesis and apoptosis, as well as carcinogenesis and tumor progression [2], [3]. Unlike other CCN family members, which encompass four distinct structural modules, WISP2 contains only three structural modules and lacks the carboxy-terminal domain [1], [4] believed to act as a potential proliferation module [5]. In non-invasive estrogen-dependent human breast cancer cells, WISP2 is induced by classical activators of cellular proliferation including estrogen, progesterone, epidermal growth factor, and insulin-like growth factor 1 [6]–[10]. We and others have proposed that WISP2 plays a dual role in the progression of breast and pancreatic cancer, acting as an oncogenic promoter at early stages of tumor development and subsequently, at later stages, as a suppressor of the invasive phenotype [11]–[13]. Additional studies have shown that WISP2 is highly expressed in less aggressive breast cancer cell lines such as MCF7, compared to non-transformed cells that express low levels of WISP2. In clear contrast, WISP2 is not detected in highly aggressive breast cancer cell lines such as MDAMB231 [13], [14] and importantly, ectopic expression of WISP2 in this cell line was accompanied by attenuation of the proliferative and invasive phenotype [13]. Similar findings were obtained when WISP2 was transcriptionally upregulated by glucocorticoids in the same cellular model [15]. In concordance with the cellular findings, the clinical data show that WISP2 expression is principally detected in preneoplastic disorders such as non-invasive ductal carcinoma *in situ* (DCIS) and atypical ductal hyperplasia, whereas WISP2 expression levels were either minimal or undetectable in invasive breast tumors [11], [16].

The epithelial to mesenchymal transition (EMT) is of critical importance in the developmental and tissue remodelling process. Accumulating evidence suggests a crucial role for EMT in cancer progression, a process associated with disruption of E-cadherin-mediated intercellular junctions and loss of several epithelial markers as well as increased expression of mesenchymal markers [17]–[19]. EMT is therefore characterized by a scattered mesenchymal phenotype and exacerbation of the invasive and metastasis potential in cancer cells. We have previously shown that WISP2 knock-down in MCF7 cells is accompanied by estrogen-independent cell growth linked to loss of estrogen receptor alpha (ERα) expression and increased expression of key components of TGF-β signaling pathway, thereby promoting EMT [16]. Furthermore, recent data indicate that WISP2 can block expression of miR-10b [20], a non-coding RNA known to play a role in invasion and metastasis [21]. Taken together, these findings suggest that loss of WISP2 is associated with breast cancer progression [22].

Besides EMT, breast cancer progression has been associated with increased “stemness”, that is a tumor phenotype with stem-like cell properties [23], [24]. We here show for the first time, that WISP2 ablation leads to the enrichment of a stem-like cell population characterized by a CD44^high^/CD24^low^ and aldehyde dehydrogenase positive phenotype, increased exclusion of Hoechst 33342, mammosphere formation and tumor formation by low-cell inoculums in nude mice. These findings suggest that WISP2 knock-down is accompanied by EMT as well as by increased stemness which may explain the impact of WISP2 on the invasiveness of breast cancer cells.

## Materials and Methods

### Cell lines

Human transformed primary embryonal kidney HEK293T and human breast carcinoma MCF7 and MDAMB231 cell lines, derived from the ATCC (American Type Culture Collections, VA, USA), were maintained in DMEM (Dulbecco modified Eagle's medium) supplemented with 10% (v/v) FBS (fetal bovine serum). MCF7-sh-WISP2 and MCF7-sh-scrambled cell lines were established by transfection of MCF7 cells with WISP2 or scrambled directed sh-RNA plasmid as described previously [13] and were maintained in DMEM (Dulbecco modified Eagle's medium) supplemented with 10% (v/v) FBS (fetal bovine serum).

### Transfections

For luciferase reporter gene assays, cells were seeded in 12-well plates and transfected with plasmids using the Fugene HD reagent (Promega). After 24 h, cells were incubated in free serum-medium for 24 h and then treated with various concentrations of TGF-β, or with conditioned medium from various cell lines, in presence or absence of SB43152, a specific inhibitor of TGF-βRII. After 16 h, luciferase activities were measured using the Dual luciferase assay (Promega).

### Real-time RT (reverse transcription)-PCR

Total RNA was extracted from all cell lines using the TRIzol® RNA purification reagent. RNA quantity and purity were determined by using a NanoDrop ND-1000. Total RNA (1 µg) from each sample was reverse transcribed and real-time RT–PCR measurements were performed as described previously [9] using an Mx3000P apparatus (Agilent) with the corresponding SYBR Green kit. PCR primers were designed with Primer 3 (Agilent). Gene expression was normalized to TBP and RPLP0 (also known as 36B4).

### Immunoblotting

Cell lysates were made in NTEN lysis buffer (0,5% Nonidet P40, 20 mM Tris/HCl pH 8, 1 mM EDTA and 150 mM NaCl) containing 1 mM PMSF, 1 µM leupeptin and aprotinin, 1 mM orthovanadate and 1 mM DTT and cleared by centrifugation (20 min at 10 000 g). Protein (50–100 µg) was separated by SDS/PAGE (7 or 10% gels) and transferred on to nitrocellulose membranes. The membranes were blocked with saturating buffer for 1 h at room temperature (25°C), then probed with the following specific antibodies: CD44, actin, LEF1, FOXC1, ZEB1, Claudin-3, Claudin-4, Claudin-7, E-cadherin, IGFBP7, Oct3/4, Nanog and WISP2 were obtained from Santa Cruz Biotechnology, ALDH1A1, ALDH2 and ALDH1A3 were obtained from Abcam, Sox9 was obtained from Aviva System Biology, SNAI2, p-Smad2, p-Smad3, Smad2/3, Smad4 were obtained from Cell Signaling Technology. Membranes were then washed and incubated with horseradish peroxidase-conjugated secondary antibodies for 2 h. Membranes were washed extensively and developed with an enhanced chemiluminescence kit (Bio-Rad) using Chemidoc system (Bio-Rad). Protein quantitation was calculated using Image Lab software developed by Bio-Rad.

### Efflux Assays

Semiconfluent cells were re-suspended in growth medium at 1×10^6^ cells per ml and 5 µg/ml Hoechst 33342 (Sigma) for 45 min at 37°C in presence or absence of 50 µM Verapamil (Sigma) in a constant-temperature water bath. Subsequently, the cells were washed in PBS containing 2% BSA, re-suspended in growth medium and incubated for 45 min at 37°C for efflux. Cells were centrifuged and mounted on a slide before photomicroscopy. Images were obtained on a station microscope Leica, Qfluoro and at least 6 fields/sample were analyzed.

### Side population (SP) and flow cytometry

The SP protocol was essentially performed as described by Goodell et al [25]. Cells (1×10^6^ cells per ml) were incubated in Dulbecco modified Eagle's medium containing 2% heat-inactivated FBS, 10 mM Hepes and 5 µg/ml Hoechst 33342 (Sigma) for 120 min at 37°C in the absence or presence of verapamil (50 µM). Propidium iodide was added to discriminate dead cells. The SP population was identified and sorted by its fluorescence profile in dual wavelength analysis (450/20 and 675/20 nm) after excitation at 350 nm. Samples were analyzed by flow cytometry using a FACS LSRII (BD Biosciences). For the determination of CD44/CD24 phenotype, cells were washed with phosphate-buffered saline (PBS), detached with accutase treatment and re-suspended in PBS supplemented with 0.5% BSA. Combinations of fluorochrome-conjugated monoclonal antibodies against human CD44 (FITC) and CD24 (APC) were obtained from Beckman Coulter. Specific antibodies or the respective isotype controls were added to the cell suspension, as recommended by the manufacturer, and incubated at 4°C in the dark for 20 min. Cells were washed with PBS containing 0.5% BSA, centrifuged and re-suspended in PBS with 2% paraformaldehyde. The labeled cells were analyzed on a FACS LSR II (BD Biosciences).

### Analysis of ALDH Activity

The ADEFLUOR® assay was performed as described by the manufacturer (StemCell Technologies). Briefly, MCF7-sh-scrambled and MCF7-sh-WISP2 cells were harvested, counted and rinsed with PBS buffer. Cells were re-suspended in ADEFLUOR® assay buffer (2×10^5^/ml) and incubated with ADEFLUOR® substrate for 45minutes at 37°C to allow substrate conversion. Half of the sample were transferred to tubes containing the specific ALDH inhibitor, diethylaminobenzaldehyde (DEAB). After centrifugation, cells were suspended in ADEFLUOR® assay buffer and ALDH activity analysis was performed by flow cytometry using a FACS LSR II (BD Biosciences).

### Mammospheres culture

MDAMB231, MCF7-sh-scrambled and MCF7-sh-WISP2 cell were plated in ultra-low attachment plate (Nunc®) in medium containing DMEM/F12 serum-free, supplemented with 100µg/ml gentamycin (Sigma-Aldrich), B27 (Life Technologies), 20 ng/ml human epidermal growth factor (EGF, Life Technologies), 20 ng/ml Human basic fibroblast growth factor (bFGF, Life Technologies) and 1% antibiotic-antimycotic solution (Life Technologies) at a density of 20,000 cells/ml. Mammosphere medium was added to plates every four days. After two weeks, they were collected, dissociated in 0.05% trypsin, 0.25% EDTA and re-seeded at 10.000 cells/ml for second passages. Enumeration of mammospheres was realized for first and second generations. The mammosphere-containing media was harvested, gently centrifuged, rinsed carefully not to disturb the sphere pellets, and re-suspended in the appropriate medium. The sphere suspensions were counted on a microscope Nikon Eclipse at X10. Results are expressed as a percentage of mammosphere forming units (%MFU) from the total number of cells plated.

### Immunofluorescence

MCF7-sh-scrambled and MCF7-sh-WISP2 were fixed with 4% paraformaldehyde in phosphate-buffered-saline, and then treated briefly with 0.1% Triton X-100 in PBS. After rinsing with PBS, cells were incubated for 1 h at room temperature with anti-fibronectin (BD Biosciences), rinsed and incubated for 1 h with Alexa Fluor 594 secondary antibody (Life Technologies). Nuclei were stained with 4′,6′-diamino-2-phenilindol (DAPI) at a concentration of 1µg/ml. Images were obtained on a station microscope Leica/Qfluoro system.

### *In vivo* tumorigenicity assay

All experiments were conducted according to the European Communities Council Directive (2010/63/UE) for the care and use of animals for experimental procedures and complied with the regulations of the French Ethics Committee in Animal Experiment « Charles Darwin » registered at the *« Comité National de Réflexion Ethique sur l'Experimentation Animale »* (Ile-de-France, Paris, no5). Ethics committee specifically approved this study. Subconfluent MCF7, MCF7-sh-scrambled and MCF7-sh-WISP2 cells were trypsinized, re-suspended in serum-free medium and mixed with an equal volume of cold Matrigel (Becton Dickinson). Cell suspension (0.2×10^6^ cells in 100 µL) was injected into the mammary fat pad of ovariectomized female Swiss nu/nu mice. The animals were weighed daily and tumor size was determined 3 times per week. At the end of the experiment, mice were anesthetized and euthanized by cervical dislocation. Tumor volumes (mm^3^) were calculated according to formula: [(length^2^ × width)/2]. Each treatment group was composed of at least 6 animals. All efforts were made to minimize suffering.

### TGF-β ELISA

For quantification of active TGF-β1 in culture supernatants, sandwich ELISA were performed using isoform-specific capture and detection antibodies (R&D Systems). For measuring the total (active + latent) levels of TGF-β1, aliquots of culture supernatants were pretreated with 1 N HCl and then neutralized with an equal volume of 1.2 N NaOH/0.5 M HEPES before the assay measurements to activate latent forms of TGF-β.

### Statistical analysis

Data are shown as the average of ±S.D. for at least three independent experiments. Differences between test and control conditions were assessed by Student's *t* test analysis. Significance is indicated by: * when p<0.05, ** when p<0.01 and *** when p<0.001.

## Results

### WISP2/CCN5 loss confers a CD44^high^/CD24^low^ phenotype

**Figure 1:**
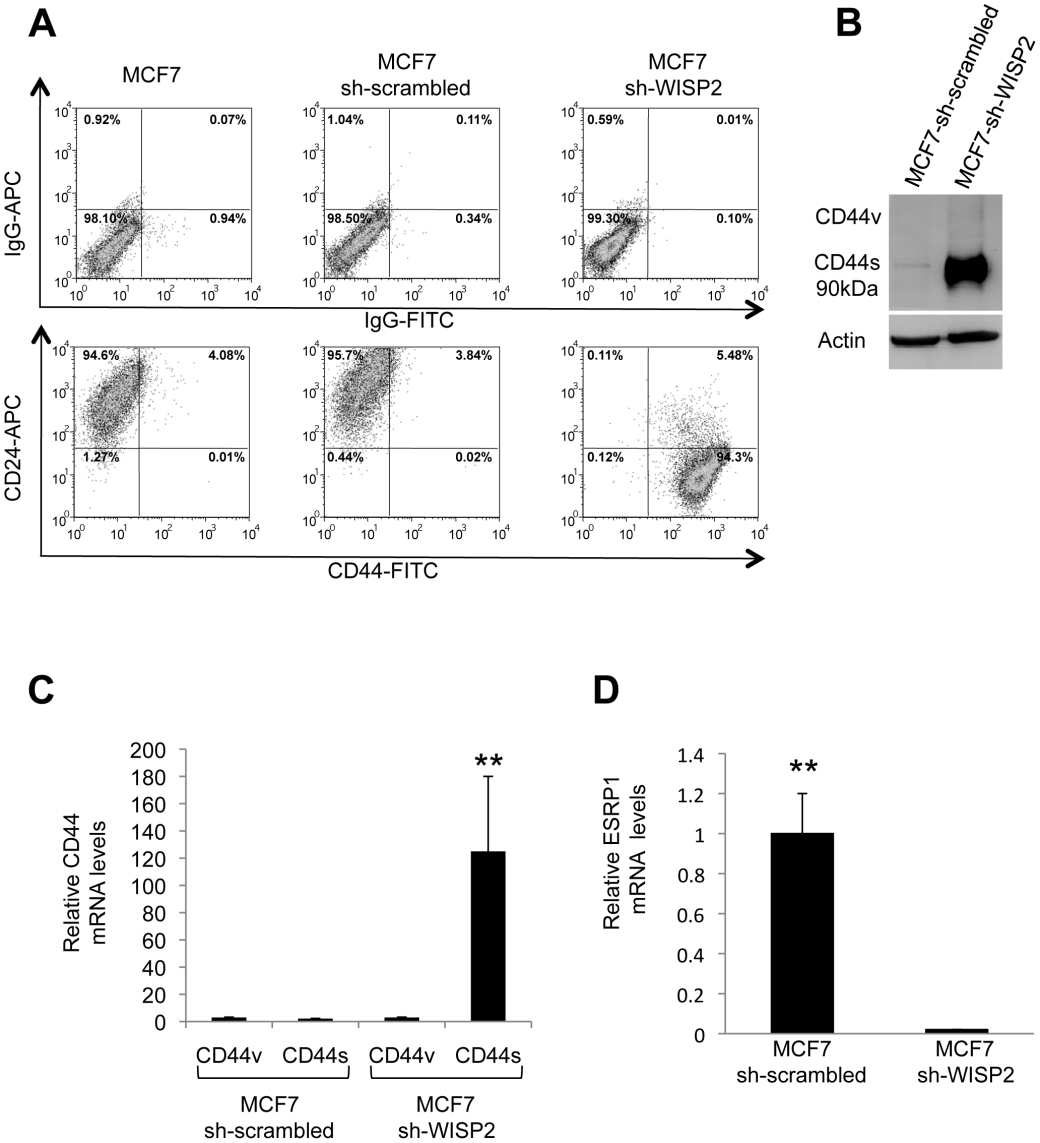
MCF7-sh-WISP2 cells predominantly express CD44^high^/CD24^low^ phenotype. (**A**) CD44 and CD24 expression profile of the MCF7, MCF7-sh-scrambled and MCF7-sh-WISP2 cells was assessed by flow cytometry, using APC-conjugated anti-CD24 and FITC-conjugated anti-CD44 antibodies. Gates are based on the isotype controls. The numbers indicate the fraction of cells present in each population. (**B**) Proteins extracted from MCF7-sh-scrambled and MCF7-sh-WISP2 cells were separated by SDS/PAGE and blotted using an antibody that recognizes all CD44 isoforms. Actin was used as loading control. (**C**) RNA was isolated from MCF7-sh-scrambled and MCF7-sh-WISP2 cells and analyzed by qRT-PCR using primers that specifically detect either CD44s or CD44v isoforms. (**D**) Expression of ESRP1 was determined by qRT-PCR. The result after normalization to 36B4 mRNA represents the relative mRNA transcripts levels and is the means ± SD of triplicate experiments. **p<0.01.

Recent findings have demonstrated that *in vitro* EMT can induce a stem-like cell phenotype [23]. We have previously shown that WISP2/CCN5 knock-down is associated with induction of the EMT phenotype in MCF7 cells [16]. To determine if WISP2/CCN5 knock-down also promotes a tumor stem-like cell phenotype, flow cytometry analysis was used to sort cells based on the expression of CD44 and CD24, two cell surface markers whose expression in the CD44^high^/CD24^low^ configuration has been associated with breast cancer stem cells [26]. The results show that MCF7-sh-WISP2 cells exhibited a higher CD44^high^/CD24^low^ cells subpopulation (94.3%) compared with the MCF7 cells (0.01%) and control MCF7-sh-scrambled cells (0.02%) as well as CD44^high^/CD24^med^ subpopulation (5.48%) (Fig. 1A). We have analyzed FACS-sorted CD44^high^/CD24^low^ and CD44^high^/CD24^med^ cells for variation of EMT markers and found no differences between these two cell populations (data not shown). CD44 can exist in a variety of isoforms due to alternative splicing, and EMT has been associated with a switch from the variant isoform (CD44v) to the standard isoform (CD44s) during breast cancer progression [27]. We therefore characterized the expression of CD44 splicing at both the protein and mRNA levels (Fig. 1B–C). Western blot analysis with an antibody that recognizes all CD44 isoforms revealed a dramatic upregulation of CD44 protein in MCF7-sh-WISP2 cells compared to MCF7 control cells (Fig. 1B). Furthermore, these cells expressed the CD44s isoform (90 kDa) that is associated with the EMT phenotype. Quantitative RT-PCR analysis of CD44 mRNA using isoform-specific primers showed that expression of the CD44v containing exons _V_3/_V_4 was decreased, while expression of CD44s was increased in MCF7-sh-WISP2 cells (Fig. 1C).

We next determined the expression of the epithelial splicing regulatory protein 1 (ESRP1) that has been implicated in CD44 alternative splicing [28]. The results show that ESRP1 expression was strongly decreased in MCF7-sh-WISP2 cells (Fig. 1D), in agreement with ESRP1 being an epithelial specific splicing factor [28]. Interestingly, the decrease in ESRP1 expression was reflected in an increase of CD44s in MCF7-sh-WISP2 cells (compare Fig. 1C and 1D).

### WISP2/CCN5 down regulation increases aldehyde dehydrogenase activity

**Figure 2:**
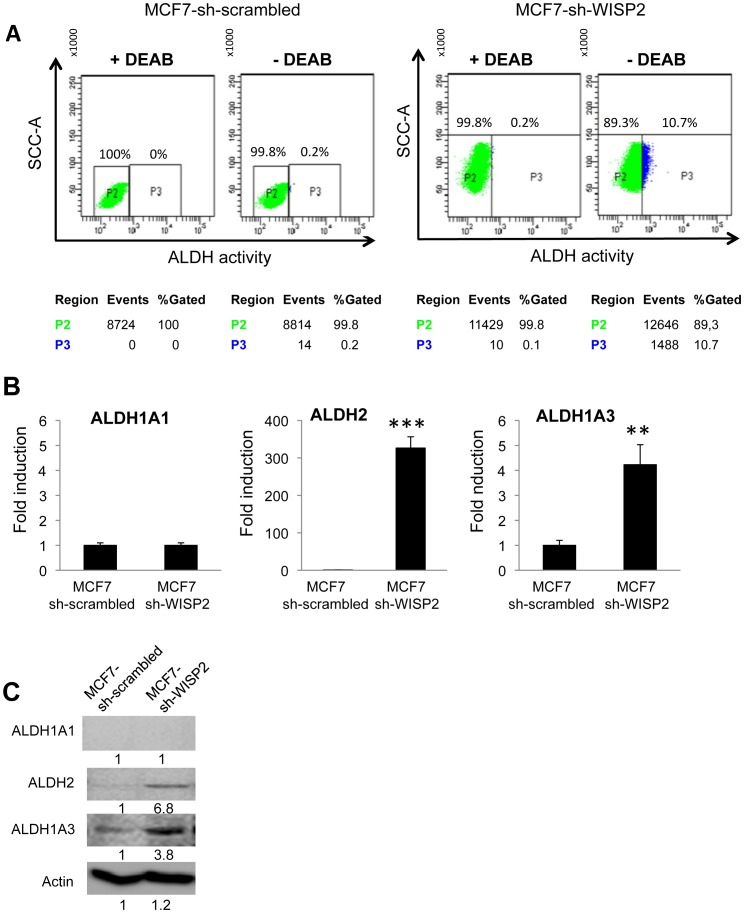
MCF7-sh-WISP2 cells show enhanced ALDH activity. (**A**) Flow cytometry analysis of ALDH activity. Cells were treated with ALDEFLUOR in the presence or absence of ALDH inhibitor DEAB followed by flow cytometry analysis. DEAB was used to establish baseline fluorescence of these cells (P2) and to define the ALDEFLUOR-positive region (P3). The numbers indicate the number of events and the fraction of cells present in each population. (**B**) Expression of different ALDH isoforms was determined by qRT-PCR. The figure indicates the expression of ALDH after normalization with 36B4 mRNA and corresponds to means ± SD of three independent experiments. Fold induction is calculated as the expression of the indicated marker in MCF7-sh-WISP2 cells compared to the expression in MCF7-sh-scrambled cells. (**C**) Expression of ALDH protein was determined by Western blotting. Actin was used as loading control. Levels of proteins were calculated by densitometry and listed beneath the bands. **p<0.01, ***p<0.001.

Aldehyde dehydrogenase (ALDH) is considered as a biomarker for stem/progenitor cells phenotype [29]. Therefore, we compared ALDH mRNA levels by real-time RT-PCR and ALDH enzyme activity by ALDEFLUOR assays in our control and WISP2 knock-down cell lines. The results show that the percentage of ALDH-positive cells is very low for the MCF7-sh-scrambled population (0.2%), whereas the fraction of ALDH-positive cells increased to almost 11% for the MCF7-sh-WISP2 cells (Fig. 2A). The specificity of ALDH activity in these cells was confirmed by sensitivity to the competitive ALDH inhibitor, DEAB. Our previous microarray analysis revealed a significantly increased in the expression of ALDH1A3 and ALDH2 mRNA in MCF7-sh-WISP2 cells [16]. To determine if the expression levels of these isoforms may explain the increased ALDH catalytic activity, mRNA was isolated from each cell line and quantitative RT-PCR performed with isoform specific primers. The results indicate that ALDH2 and, to a lesser extent, ALDH1A3 expression correlated with the ALDEFLUOR activity of the MCF7-sh-WISP2 cells (Fig. 2B). Subsequent Western blot analysis confirmed that loss of WISP2 was accompanied by increased expression of both ALDH2 and ALDH1A3 while ALDH1A1 was undetectable in both cell lines (Fig. 2C). This result suggests that ALDEFLUOR activity in breast MCF7-sh-WISP2 cells is not due to the activity of ALDH1A1, but likely to other isoforms including particular ALDH2 and/or ALDH1A3.

We observed considerable discrepancy between the expression levels of ALDH2 mRNA and protein suggesting that post-translational mechanisms may contribute to the regulation of ALDH2 protein expression in MCF7-sh-WISP2 cells.

### WISP2/CCN5 down regulation increases mammospheres formation

**Figure 3:**
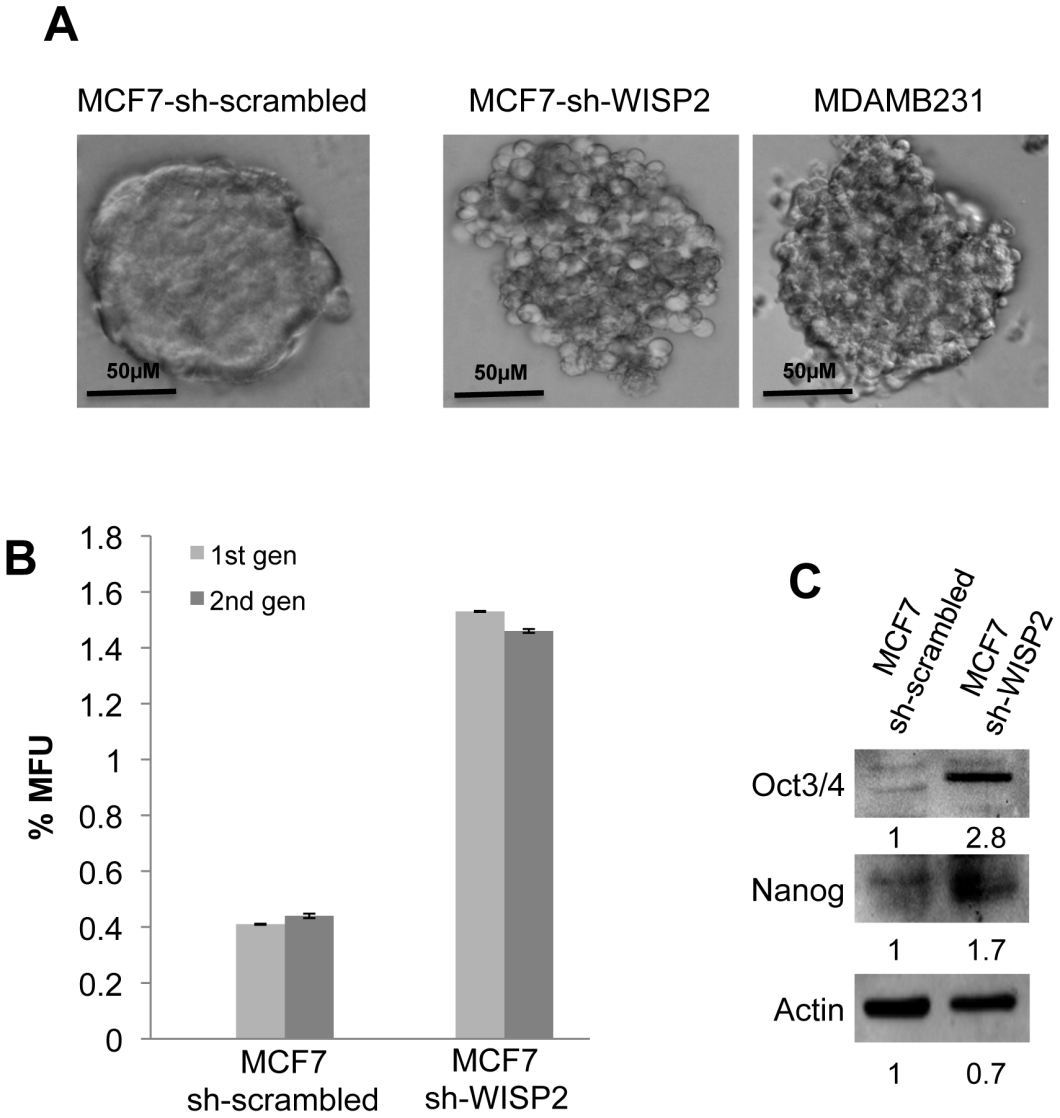
Mammosphere formation and self-renewal of WISP2-downregulated MCF7 cells. (**A**) MCF7-sh-scrambled, MCF7-sh-WISP2 and MDAMB231 cell lines were plated in low-serum non-adherent culture conditions at a density of 20,000 cells/ml. Images were obtained by microscopy at ×10 magnification and are representative of the mammosphere formed after seven days in culture. (**B**) Mammospheres were counted after seven days culture (1^rst^ generation) followed by dissociation using trypsin and repassaged at density of 10,000 cells/ml in mammosphere medium. Mammosphere formation was then estimated after seven days in culture (2^nd^ generation). Results are calculated as a percentage of mammosphere forming units (%MFU) from the total number of cells plated and are representative of three independent experiments. (**C**) Protein extracts of MCF7-sh-scrambled and MCF7-sh-WISP2 mammospheres were tested by Western blotting for Oct3/4 and Nanog expression. Actin was used as a loading control. Levels of proteins were calculated by densitometry and listed beneath the bands.

Since mammosphere formation is considered to be a characteristic of cancer stem cells, we compared the ability of MCF7-sh-scrambled and MCF7-sh-WISP2 cells to form mammospheres in culture. The two cell lines formed mammospheres of distinct size and morphology (Fig. 3A). Specifically, the ER-positive MCF7-sh-scrambled cells forming mostly uniform and regular colonies while the ER-negative MCF7-sh-WISP2 cells forming loose, irregular colonies similar to MDAMB231 cells [30], [31]. To test the self-renewal capability of the mammosphere-forming cells, the primary mammospheres were dissociated into single cells and used for a secondary mammosphere assay. We observed a significantly higher number of primary mammospheres with MCF7-sh-WISP2 cells (∼4-fold) compared to MCF7-sh-scrambled cells. Moreover, the proportion of mammosphere-forming cells isolated from the primary mammospheres remained the same for both MCF7 sub-cell lines (Fig. 3B). Importantly, Western blot analysis indicated that spheres formed from MCF7-sh-WISP2 expressed the stem cell markers Oct3/4 and Nanog (Fig. 3C). This functional assay indicates that the mammosphere-forming WISP2-silenced cells showed a less-differentiated state.

### MCF7-sh-WISP2 cells show increased efflux of Hoechst 33342

**Figure 4:**
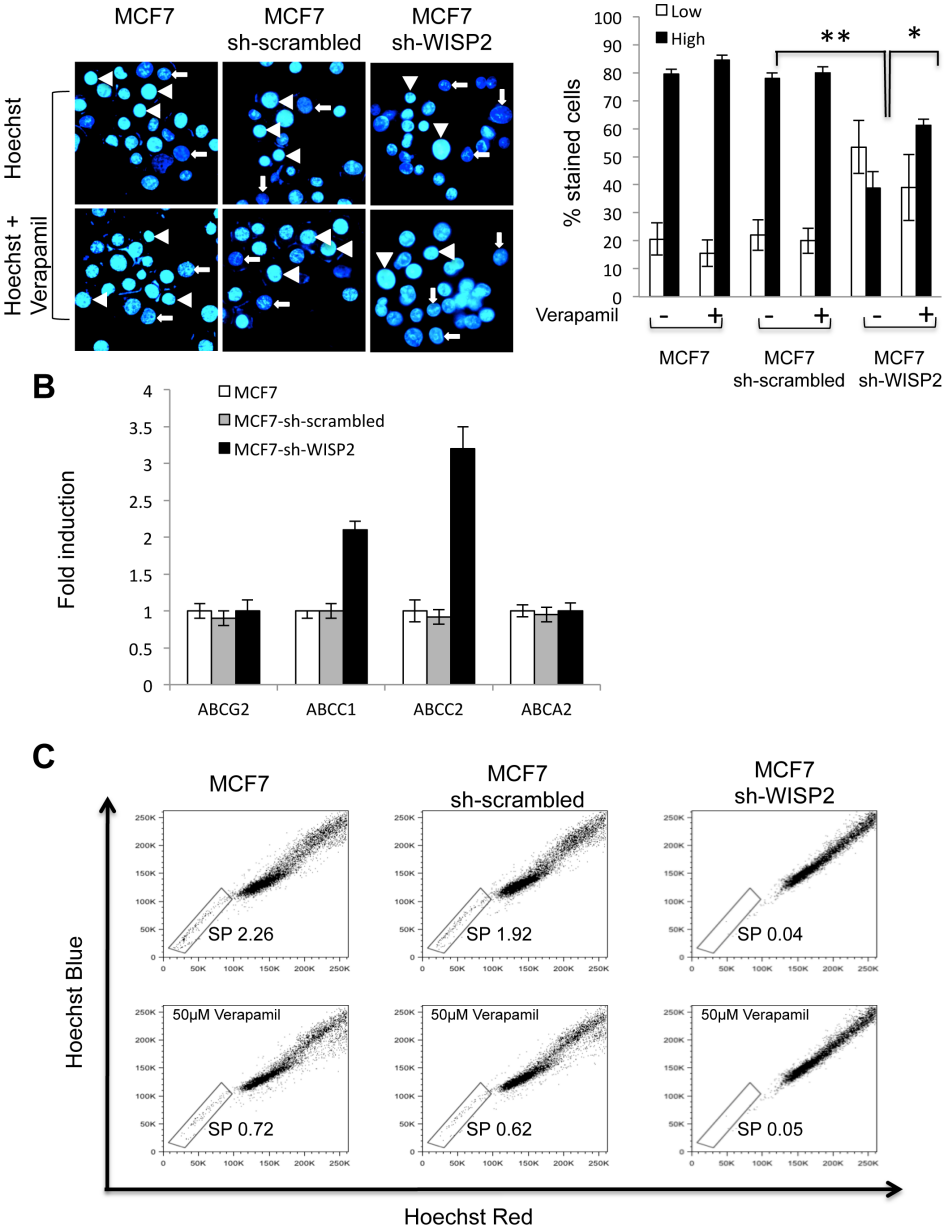
Efflux of Hoechst 33342 by MCF7, MCF7-sh-scrambled and MCF7-sh-WISP2 cells. (**A**) MCF7, MCF7-sh-scrambled and MCF7-sh-WISP2 cells were exposed to Hoechst 33342 in the presence or absence of Verapamil, a non-specific inhibitor of membrane transporters. Images were obtained by microscopy at 40× magnification. Arrows indicate low staining and arrowheads high staining. The histogram indicates the quantification of low and high Hoechst-stained cells, in presence or absence of Verapamil, expressed as (%) of total counted cells. (**B**) RNA was isolated from MCF7, MCF7-sh-scrambled and MCF7-sh-WISP2 cells and the expression of selected ABC transporters including ABCG2, ABCC1, ABCC2 and ABCA2 was analyzed by qRT-PCR. Fold induction in mRNA expression was calculated as compared with the corresponding mRNA in MCF7-sh-scrambled cells. Results are the means ± SD of three independent experiments. (**C**) The side-population (SP) cells were analyzed by Hoechst and flow cytometry. After staining with H33342, in presence or absence of Verapamil, Hoechst Blue and Hoechst Red fluorescence were measured by flow cytometry. Data are representative of three individual analyses. **p<0.01, ***p<0.001.

A characteristic shared by many adult stem cells is the ability to efflux Hoechst dyes due to the elevated expression of ATP-binding cassette (ABC) transporters on the cell surface [32]. The ability of MCF7, MCF7-sh-scrambled and MCF7-sh-WISP2 cells to extrude Hoechst 33342 dye was determined by fluorescence microscopy, as shown in Figure 4A. We observed two distinct cell sub-populations that we named low and high. Low appears as Hoechst blue-pale, while high shows intense staining. A significantly smaller fraction of MCF7-sh-WISP2 cells (38.9%) shows high staining compared to MCF7 cells (84.4%) and MCF7-sh-scrambled cells (80%). In the presence of verapamil, a non-specific inhibitor of membrane transporters, an increased fraction (61%) of MCF7-sh-WISP2 cells shows intense staining, indicating the presence of functional drug transporters. To determine the factors responsible for the efflux of Hoechst 33342, real-time qRT-PCR was used to quantify mRNA expression of various ABC transporters in MCF7-sh-WISP2, MCF7-sh-scrambled and MCF7 cells. The results revealed a significant increase of ABCC1 and ABCC2 expression in WISP2-silenced cells (2 and 3.3-fold higher expression, respectively, than the MCF7 and MCF7-scrambled cells) (Fig. 4B), suggesting that ABCC1 and ABCC2 contribute to the increased efflux of Hoechst 33342 in these cells.

As side-population (SP) cells have a high efflux capacity owing to functional expression of ABC transporters, we quantified SP cells within MCF7, MCF7-sh-WISP2 cells and MCF7-sh-scrambled cells. Following staining with Hoechst 33342, cells were analyzed by flow cytometry. When Hoechst blue and red fluorescence are plotted against one another, cells, that have excluded the dye, form a tail-like structure called SP, that separates these cells from the majority of brightly staining mature tumor cells (non-SP cells) [25]. Parental MCF7 cells showed a distinct SP, accounting for 2.26% of the whole population, confirming that this established cell line contains at least 2 phenotypically distinct sub-populations, one of which displays stem cell features [33]. Similarly to MCF7 cells, MCF7-sh-scrambled cells displayed a significant SP (1.92%), in marked contrast to the MCF7-sh-WISP2 cells that show a very low fraction of SP cells (0.04%). In MCF7 and MCF7-sh-scrambled, the fraction of SP cells were consistently diminished by addition of verapamil (Fig. 4C), confirming that the side population cells are caused by Hoechst efflux.

### Tumorigenicity of MCF7-sh-WISP2 cells in mice

**Figure 5:**
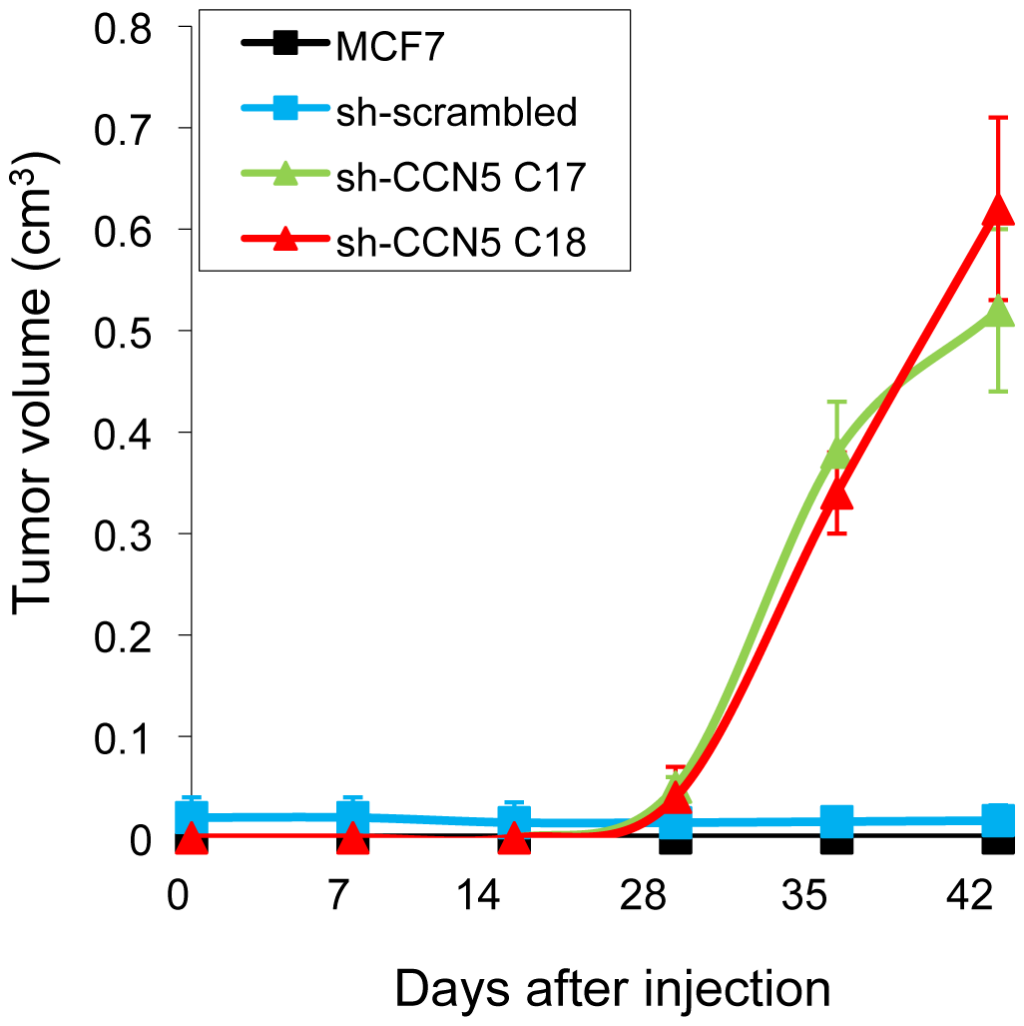
Tumorigenicity of MCF7-sh-WISP2 xenografts. Orthotopic tumors were induced by injecting 200.000 MCF7 and MCF7-sh-scrambled as well as two independent clones of MCF7-sh-WISP2 cells (C17 and C18) into the mammary fat pad of nude mice and the tumor growth was followed for 42 days.

MCF7 cells are weakly tumorigenic in athymic nude mice and require estrogen supplementation to generate a tumor [34]. We have previously reported that WISP2 knock-down in MCF7 cells was associated with the acquisition of estradiol-independent growth due to loss of *ESR1* mRNA gene expression [13]. We therefore examined whether the MCF7-sh-WISP2 cells would be able to form tumors in nude mice without estrogen supplementation. To establish orthotopic tumors, MCF7 and MCF7-sh-scrambled cells as well as two independent WISP2-silenced clones, were injected into the mammary fat pad of nude mice. After 7 weeks in the absence of exogenous estrogen supplementation, mice injected with as few as 0.2×10^6^ MCF7-sh-WISP2 cells formed large tumors in clear contrast to the MCF7 and MCF7-sh-scrambled cells (Fig. 5). These results suggest that WISP2 knock-down increased the tumorigenicity of MCF7 cells.

### MCF7-sh-WISP2 cells express both EMT and stem-like cells markers

**Figure 6:**
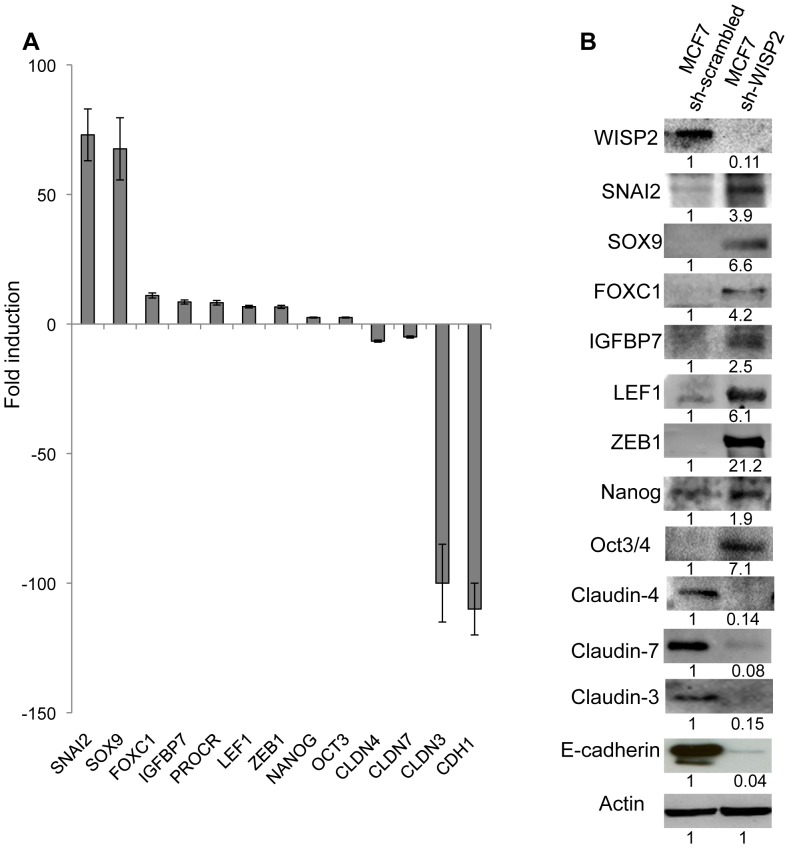
MCF7-sh-WISP2 cells express both EMT and stem cells markers. The expression of EMT and stem cell markers were compared between MCF7-sh-scrambled and MCF7-sh-WISP2 by (**A**) qRT-PCR. Fold induction is calculated as the expression of the indicated marker in MCF7-sh-WISP2 cells compared to the expression in MCF7-sh-scrambled cells. (**B**) Western-blot analysis. Actin was used as a loading control. Levels of proteins were calculated by densitometry and listed beneath the bands.

To determine whether these cells present gene expression characteristics described for breast cancer stem cells, we selected genes identified by microarray analysis [16] and confirmed differentially expressed genes by Western blotting, and PCR analysis (Fig. 6A, B). We found a strong increase in the expression of SNAI2 (∼73-fold) and SOX9 (∼67-fold) which may both contribute to the maintenance of human breast cancer stem cell phenotype [35]. There was also a considerable increase in the expression of others genes described as stem cell markers and expressed in CD44^high^ fractions of normal and breast cancer tissues [36], including FOXC1(∼11-fold), IGFBP7 (∼8.5-fold), PROCR (∼8.2-fold), LEF1 (∼6.7-fold) and ZEB1 (∼6.6-fold). In addition, we observed that MCF7-sh-WISP2 cells have considerably reduced expression of CLDN3 (∼100-fold), CLDN4 (∼6.6-fold), CLDN7 (∼5-fold) as well as CDH1 (∼110-fold) genes. Although the levels of mRNA (Fig. 6A) and protein (Fig. 6B) varied between the various markers, the variation was always in the same direction (i.e. increasing or decreasing), for the mRNA and protein. The variable correlation between mRNA and protein levels can be explained by additional regulation of mRNA processing at the posttranscriptional and posttranslational level [37], [38].

These results provide further indications that the MCF7-sh-WISP2 cells display both increased expression of stem cell markers as well as decreased expression of claudin proteins.

### TGF-β activation in WISP2-silenced cells

**Figure 7:**
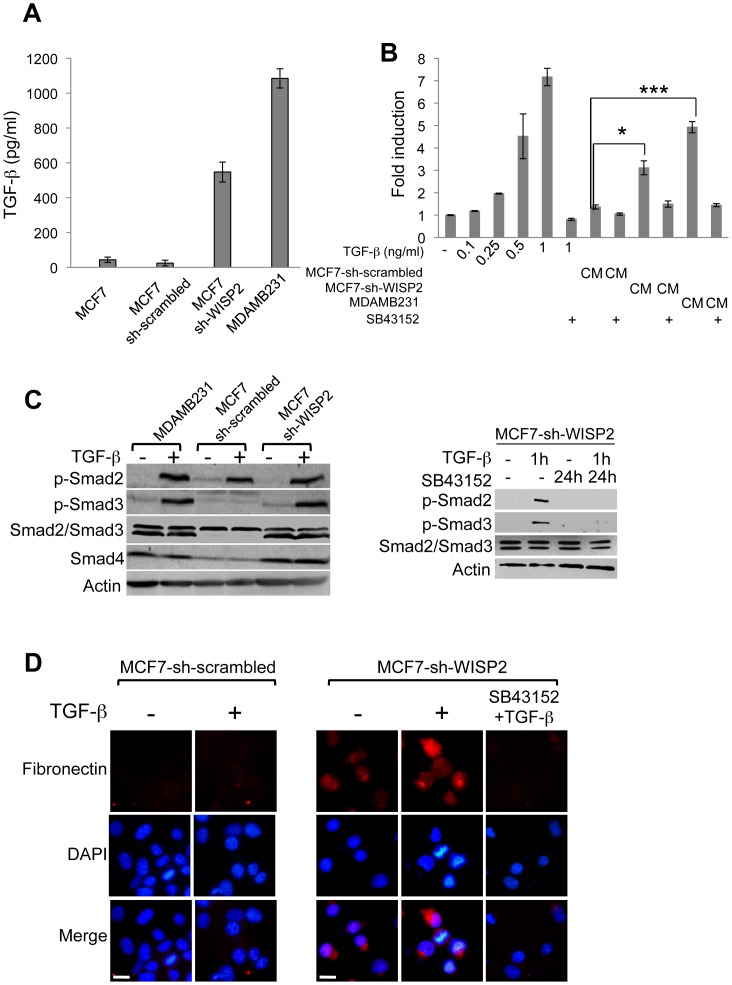
WISP2 knock-down is accompanied by enhanced TGF-β signaling. (**A**) Quantification by sandwich ELISA of total (active plus latent) TGF-β1 in conditioned medium from MCF7, MCF7-sh-scrambled, MCF7-sh-WISP2 and MDAMB231 cells. Results are the means ± SD of triplicate experiments. (**B**) HEK293T cells were transfected with 0.3 µg of CAGA_9_-Luc and 10 ng of Tk-renilla as an internal control. Cells were treated with various concentrations of TGF-β (0 to 1 ng/ml) or conditioned medium (CM) from MCF7-sh-scrambled, MCF7-sh-WISP2 and MDAMB231 cells treated or not with SB43152 (10^−6^M), a specific inhibitor of TGF-β receptor kinase, for 16 h. The fold induction was determined relative to the activity of the reporter alone and represents three independent experiments assayed in triplicate. (**C**) Cells were treated with or without TGF-β (2 ng/ml) for 1 h, in the presence or absence of SB43152 (10^−6^M), for 24 h. The protein levels of Smad2, Smad3, Smad4, p-Smad2 and p-Smad3 were examined by Western blotting using the corresponding antibodies. Actin was used as a loading control. (**D**) Immunofluorescence analysis of fibronectin expression was performed for MCF7-sh-scrambled and MCF7-sh-WISP2 cells. Cells were treated as described in (C), and were then fixed, permeabilized and immunostained with anti-fibronectin and Alexa Fluor 594-conjugated secondary antibodies, as described in Materials and Methods. Nuclear DNA was stained with DAPI. Images were obtained by microscopy at ×40. Scale bars, 10 µm. *p<0.05, ***p<0.001.

TGF-β signaling plays an important role in regulating the pluripotency of human embryonic stem cells [39] and is also involved in EMT [40]. Furthermore, we have previously shown that both TGF-βRII mRNA and protein levels are increased in MCF7-sh-WISP2 cells [16]. For these reasons, the influence of TGF-β signaling was examined in further detail. For comparison, we included the MDAMB231 cell line which is known to be responsive to TGF-β1 but is resistant to growth inhibition activity [41]. Both MDAMB231 and MCF7-sh-WISP2 cells express CD44 and TGF-β receptors. The expression of TGF-β1 mRNA was increased in MCF7-sh-WISP2 cells compared to MCF7-sh-scrambled cells (data not shown). TGF-β isoform-specific sandwich ELISA showed a significant increase in total (latent + active) TGF-β1 in conditioned medium from MCF7-sh-WISP2 cells (547 pg/ml) compared to MCF7 or MCF7-sh-scrambled cells (43 and 24 pg/ml respectively) (Fig. 7A). To investigate whether MCF7-sh-WISP2 cells generated active TGF-β1, HEK293T cells were transfected with (CAGA)_9_-luc, a TGF-β specific reporter construct and were stimulated with conditioned medium from MCF7-sh-scrambled or MCF7-sh-WISP2 cells. Conditioned medium from MDAMB231 cells was used as a positive control given that these cells expressed high levels of TGF-β1 (1085 pg/ml) (Fig. 7B). Stimulation of the reporter was observed with conditioned medium of MCF7-sh-WISP2 cells. Importantly, conditioned medium obtained from MCF7-sh-WISP2 cells treated with SB43152, a TGF-βRI inhibitor, did not stimulate the reporter (Fig. 7B). This stimulation could be compared to that observed with a TGF-β concentration between 0.25 and 0.5 ng/ml (Fig. 7B). We further show that TGF-β signaling is activated in MCF7-sh-WISP2 cells in response to TGF-β, which can be inhibited by SB43152, as determined by the phosphorylation of SMAD2/3 (Fig. 7C). MCF7-sh-WISP2 and MDAMB231 cells also display a clear increase in the expression and phosphorylation of SMAD3, in contrast to MCF7-sh-scrambled cells (Fig. 7C).

TGF-β is a potent inducer of fibronectin on both the mRNA and protein levels [42], [43]. Immunofluorescence analysis indicated that MCF7-sh-WISP2 cells show increased expression of fibronectin in the absence of exogenous TGF-β, which is further reinforced in the presence of TGF-β and totally inhibited by SB43152 (Fig. 7D). These results clearly indicate that the TGF-β pathway is specifically and endogenously activated in MCF7-sh-WISP2 breast cancer cells.

## Discussion

A better mechanistic understanding of tumor progression and metastasis remains a major fundamental and clinical challenge. One major mechanism associated with the invasive phenotype of epithelial cancers is the epithelial to mesenchymal transition (EMT) [44]. Furthermore, studies with immortalized human mammary epithelial cells suggest that a mesenchymal phenotype may be accompanied by acquisition of stem cell-markers [23], [24].

We have previously reported that the loss of *WISP2/CCN5* gene expression in MCF7 cells is accompanied by EMT [13]. Here, we show for the first time that loss of WISP2 expression directs cells from a differentiated state toward a less differentiated and stem-like state.

The most widely accepted markers to identify mammary cancer stem cells are the expression of CD44/CD24 and ALDH [26], [29]. In particular, the cell surface protein CD44 is a major marker for stem-like cancer cells and is also expressed in metastatic cancer cells [45]. We show that loss of WISP2 expression leads to a dramatic increase in the fraction of MCF7 cells expressing the CD44^high^/CD24^low^ phenotype. CD44 exists in different splice forms that are differentially regulated during EMT, resulting in a switch in expression from the variable exon containing CD44v isoform to the standard isoform, CD44s, that is devoid of all CD44 variable exons [27]. Our results reveal that MCF7-sh-WISP2 cells express high levels of CD44s isoform while MCF7-sh-scrambled cells do not express any detectable isoforms, which might, at least in part, be due to down-regulation of the splicing regulatory protein 1 (ESRP1).

Recent evidence suggested that CD44^high^/CD24^low^ is not an absolute marker of breast cancer stem cells [46] and additional markers may be needed to classify cancer stem cells. Among these, ALDH1 has been proposed as a typical marker for breast cancer stem cells [47]. Unexpectedly, we found that the expression of ALDH2 and ALDH1A3, rather than ALDH1A1 contributed mainly to the Aldefluor activity of the MCF7-sh-WISP2 cells. Although initially surprising, our findings are coherent with a recent analysis in tumor samples from breast cancer patients showing that ALDH1A1 expression is not the primary determinant of ALDH activity as initially believed [48]. Our results are consistent with previous reports showing that ALDH2 could be a potential marker for breast cancer stem cells [48], [49].

Apart from analysis of stem cell markers, the ability of tumor cells to form mammospheres efficiently in culture is considered as a hallmark of the cancer stem cell phenotype. Our results show that MCF7 cells that have lost WISP2 expression are capable of forming loose and irregular mammospheres under ultra-low attachment conditions in serum-free media. Similar mammosphere morphology has been previously observed for MCF7-overexpressing the EMT factor Twist, suggesting that Twist may promote the stem-like cell phenotype [50]. In agreement, our previous results show that the expression levels of TWIST mRNA and protein were increased in MCF7-sh-WISP2 cells [51].

One of the pathways found specifically activated in WISP2-silenced breast cancer cells is the TGF-β signaling pathway [16]. Thus, we have identified a functional autocrine TGF-β1 signaling signature of TGF-β1/TGF-βRII/Smad activation that can be induced by increase of active TGF-β1 concentration. The TGF-β signaling pathway is known to play an important role in human embryonic stem cells as well as in tumorigenesis [39], [52]. Interestingly, many of the stem cell markers induced in MCF7-sh-WISP2 cells are known to be regulated by TGF-β. For example, we observed a high expression of Nanog and Oct3/4 in MCF7-sh-WISP2 cells; which is in agreement with previous results showing that Nanog is a direct target of TGF-β-mediated SMAD2/3 signaling in stem-like cells [53]. Similarly, TGF-β1 is a potent inducer of IGFBP7 [54] and IGFBP7 is described as a stem cell marker expressed in CD44^high^ but not in CD24^high^ cell fractions of normal and breast cancer tissues [36]. FOXC1 is highly expressed in CD44^high^ cells and regulated by TGF-β and Hedgehog, both of which are important regulators of stem cell function [55]. SNAl2 and ZEB1 are also well known TGF-β1-induced genes. We here observed increased TGF-β1 in MCF7-sh-WISP2 conditioned medium culture, which is correlated with increased SMAD3 expression and phosphorylation. SMAD3 is associated with EMT *via* the canonical TGF-β pathway. In addition, TGF-β induces SMAD3-dependent transcription in stem cells [56]. Therefore, activation of the TGF-β pathway is coherent with both the EMT phenotype and the increased stemness of the WISP2-silenced cells.

Global messenger RNA expression analysis of human breast cancers have established five “intrinsic” molecular subtypes: luminal A, luminal B, Basal-like, HER2-enriched, and the recently characterized claudin-low subgroup [57]. The claudin-low subtype exhibits low expression of genes responsible for cell-cell adhesion, particularly low expression of many of the claudin genes, including 3,4, and 7, and enrichment of markers linked to stem cell function and EMT. We have shown that loss of WISP2 expression in breast cancer cells leads to acquisition of the CD44^+^/CD24^−^ phenotype, ALDH^+^ expression, upregulation of the ABCC2 drug efflux pump expression known to be associated with less differentiated cells and stem cells [58], although they lack SP cells. Interestingly recent studies show that SP cells were more prevalent in the luminal subtype of breast cancers compared with the basal subtype while, HER2 expression was significantly correlated with the occurrence of SP cells [59]. We have shown previously that loss of WISP2 expression leads to a triple negative phenotype ER^−^/PR^−^/HER2^−^ [51]. In agreement, previous data using MDAMB231 cells, identified as a claudin-low cell line [57], have shown that this cell line do not exhibit SP in spite of a prominent CD44^+^/CD24^−^ phenotype and increased ALDH activity [59]. These results suggest that WISP2-silenced MCF7 model is representative of the claudin-low molecular subtype of the basal subfamily of breast cancer [57], [60].

In conclusion, our results demonstrate that loss of WISP2 expression in breast cancer cells is accompanied by both EMT induction and increased stemness. The generation of breast cancer stem-like cell phenotype may facilitate the identification of novel biomarkers and target discovery for the development of more efficient therapies toward the claudin-low subtype of triple negative breast cancer.
